# Cariprazine's Potential in Improving Social Dysfunction in Patients With Schizophrenia: A Perspective

**DOI:** 10.3389/fpsyt.2022.868751

**Published:** 2022-04-27

**Authors:** Petr Morozov, Roman Bekker, Youri Bykov

**Affiliations:** ^1^Pirogov Russian National Research Medical University, Moscow, Russia; ^2^Ben-Gurion University of the Negev, Beersheba, Israel; ^3^Stavropol State Medical University, Stavropol, Russia

**Keywords:** schizophrenia, cariprazine, antipsychotic, social dysfunction, D_3_ receptors

## Abstract

Social dysfunction is one of the most debilitating aspects of schizophrenia. Treatment of this complex phenomenon, constituted by negative, cognitive, and affective symptoms, has been difficult with the available pharmacological agents, hence it represents an unmet medical need. Cariprazine, a novel, third-generation antipsychotic with a unique mechanism of action has been proven to sufficiently alleviate negative, cognitive, and affective symptoms of schizophrenia. These characteristics make this compound a valid candidate for addressing social dysfunction too. In this perspective, we argue that cariprazine can be viewed as a “socializing drug” that has the ability to improve the patient's functionality and ultimately their quality of life. Data from animal research, clinical trials, an observational study, and patient cases are provided.

## Introduction

Schizophrenia is a chronic mental disorder, affecting about 1% of the population worldwide (1). First described by Eugen Bleuer (2), it is characterized by three main symptom domains: positive symptoms such as hallucinations and delusions, negative symptoms including anhedonia and blunted affect (3), as well as cognitive symptoms like deficits in executive functioning and memory problems (4). In his original concept, Bleuer also included autism as one of the main symptoms of schizophrenia (2), thus emphasizing the high prevalence of social dysfunction in the disorder, as well as its importance in determining the level of disability in everyday functioning (5). Indeed, schizophrenia is one of the most debilitating disorders with high burden of disease, which translates into around 13.4 million years lived with disability (YLDs), which is equivalent to 1.7% of total YLDs (6). Even though the advent of antipsychotic medications brought improvement in the management of schizophrenia, poor social functioning still represents an unmet medical need (7). Cariprazine, a novel, third generation antipsychotic drug might be able to bring change to this notion as it has a unique mechanism of action compared to the other medications (8).

In this perspective, we argue that cariprazine can be viewed as a “socializing drug” in the treatment of schizophrenia. To prove this, first, we provide an overview of the characteristics, impact, and background of social dysfunction in schizophrenia and then highlight data from clinical trials and real-life experiences that show cariprazine's potential to improve this aspect of the disorder.

## Social Dysfunction in Schizophrenia

Social dysfunction is a complex phenomenon that affects many different aspects of the lives of the patients including social interactions, everyday activities, and employment status (9). It has already been described as a core feature of schizophrenia in one of the earliest descriptions of the disorder, Dementia Praecox, by Kraepelin (10, 11). Social dysfunction is part of the class description of Schizophrenia Spectrum and Other Psychotic Disorders in the Diagnostic and Statistical Manual 5th Edition as well, which describes it as “For a significant portion of the time since the onset of the disturbance, level of functioning in one or more major areas, such as work, interpersonal relations, or self-care, is markedly below the level achieved prior to the onset” (12).

Research has shown that social dysfunction is primarily, but not exclusively, related to cognitive impairments of schizophrenia (9). Indeed, among the eight separable domains of cognitive impairment identified by the NIMH-Measurement and Treatment Research to Improve Cognition in Schizophrenia (MATRICS) consensus one is deficits in social cognition, which is defined as the inability to construct representations of self, others, and interpersonal interactions (13, 14). Such disturbances in social cognition compromises everyday functioning by impairing the mental operations that underlie social behavior such as being able to understand and interpret another person's emotions and intentions (15). A meta-analysis by Fett and colleagues found social cognition to be strongly associated with community functioning e.g., independent living skills or work functioning (15).

In addition to cognitive symptoms, negative symptoms have also been reported to be involved in social dysfunction in schizophrenia (16). According to a consensus, negative symptoms are comprised of five constructs: blunted affect, alogia, anhedonia, asociality and avolition (3, 17). Asociality, defined as reduction in social initiative due to decreased interest in forming close relationships with others, contributes greatly to social dysfunction in schizophrenia (17, 18). Furthermore, avolition, the reduced initiation and persistence of goal-directed activity has also been implicated as important factor in social functioning (17, 18). To give an example, in a study involving 149 patients with schizophrenia and 143 healthy controls, the severity of avolition in patients predicted the proportion of time they spent in structured and unstructured social contexts (18).

## The Impact of Social Dysfunction on Everyday Functioning

Social dysfunction is often labeled as one of the most debilitating characteristics of schizophrenia as it has a profound effect on both everyday functioning and quality of life (15, 19). It impacts education, functioning in the work environment, conduction of activities such as shopping, interpersonal relationships as well as living circumstances. For instance, findings of a study conducted in the USA reported only 18% of patients with schizophrenia to live independently (20). Similarly, another study in Singapore found 79% of patients to live with family or spouse, and reported that the percentage of patients living independently increased with age (21).

Regarding employment, about 10% of working-age patients with schizophrenia are employed in contrast to the general population, where employment rate is around 68% in the 20–29-year-old age group and 84% in the 40–49-year-old age group (22, 23). This is highly related to time of onset as well, as those who receive their diagnosis between ages 15 and 25 are more likely to be unemployed (24). Interestingly, there are indirect effects of schizophrenia on employment and education as well; children whose parents are diagnosed with schizophrenia were found we have higher odds of not graduating from primary education (25).

Another study examining the degree of dysfunction in different daily activities in people with schizophrenia found that handling medications, shopping, preparing food as well as handling finances and doing the laundry is highly difficult for patients to execute (26). In fact, only 2% of the study sample were completely independent in their daily activities (26).

Finally, in an Australian national survey involving almost two thousand patients, loneliness was reported by 80% of those affected by psychosis (27). In addition, loneliness was also identified as one of the major challenges that hinders recovery (27, 28). Importantly, loneliness is a risk factor for poorer overall cognitive performance and faster cognitive decline as well (29).

## Background of Social Dysfunction in Schizophrenia

Impaired functioning of the so-called social brain i.e., brain regions that are known to be involved in social cognition, has been described in schizophrenia patients repeatedly (30). These include changed activation of the medial and inferior prefrontal cortex as well as the hypo-activation of the amygdala (30). In addition, abnormal activity in the mirror neuron system, an important neuronal function responsible for understanding the intentions of others, has also been reported in patients with schizophrenia (31).

Besides these brain regions, neurotransmitters such as dopamine and serotonin have also been investigated to understand the potential mechanisms behind social dysfunction. Regarding the dopaminergic system, the role of D_2_ and D_3_ receptors are of particular interest given the fact that alterations in dopamine levels are regarded as a core aspect of schizophrenia (32). Indeed, D_3_ receptors agonism was reported to impair some aspects of cognitive function including social recognition and executive function, while antagonism is suggested to enhance cognition through inducing changes in the prefrontal cortex and hippocampus (33).

In terms of the serotonergic system, aggression and impulsive behavior (key determinants of social dysfunction) have been repeatedly associated with reduced serotonergic function (34). Evidence from Non-human primates indicate that the pharmacological reduction of serotonin promotes aggressive behavior while the blockade of serotonin reuptake has the opposite effect (34). This has been reported in a double-blind cross-over human study as well, where the effect of a selective serotonin reuptake inhibitor was investigated in chronically violent patients with schizophrenia (35). In fact, the results showed significant reduction in the frequency of aggressive actions without deterioration of mental state (35).

## Difficulties of Improving Social Dysfunction in Schizophrenia

Although antipsychotics, the first-line treatment for schizophrenia, are able to reduce some symptoms of the disorder, evidence regarding their effect on social dysfunction is rather limited and inconclusive (36). This also stems from the fact that there is considerable heterogeneity in how social dysfunction is defined and measured, as well as that many of the studies are neither adequately powered nor randomized (36).

### First-Generation Antipsychotics

First-generation or typical antipsychotics (FGAs) such as haloperidol are dopamine antagonists that induce considerable side effects including extrapyramidal motor symptoms (EPS) and tardive dyskinesia (TD) (37). Effects of FGAs on social dysfunction are mixed. Some studies found significant improvement in facial affect recognition in patients treated with FGAs (38, 39), while others could not report any positive change (40). Furthermore, a study conducted in 2021 reported an association between antipsychotic-induced EPS and social cognition in patients with schizophrenia, with those affected by EPS scoring worse on the different social cognition measures (37). The study also highlighted that about half of the patients treated with FGAs experienced EPS, while this number was 25% in patients treated with second-generation antipsychotics (SGAs) (37).

### Second-Generation Antipsychotics

In contrast to FGAs, SGAs or atypical antipsychotics such as risperidone are not only dopamine but serotonin antagonists as well and exhibit high affinity for 5HT_2A_ receptors (37). Given these properties many argued that SGAs may have a positive impact on social functioning (38, 39). In addition, such agents have lower incidents of EPS-like symptoms, however other adverse effects like weight gain is common (40, 41).

Although several studies assessed the effect of SGAs on different aspects of social dysfunction, positive outcomes were rather scarce (36). For instance, Bellack and colleagues compared risperidone and clozapine in terms of their ability to improve social skills after 16–29-week treatment and while improvement in general symptomatology was detected, no significant impact on social competence was found (42). Similarly, two randomized studies investigating risperidone, quetiapine and olanzapine draw the conclusion that SGAs are unable to significantly improve social cognition (43, 44). Importantly, the Clinical Antipsychotic Trials for Intervention Effectiveness (CATIE) trial, also failed to detect significant improvement in emotion perception after 2 months of treatment with SGAs (45).

In contrast to the previous results however, Fakra et al. found risperidone to be superior in a facial affect discrimination task in comparison with an FGA, haloperidol, after 4 weeks (46). Animal studies also reported similar results; clozapine was able to attenuate reduction of social behavior in mice whereas haloperidol failed to do so (47).

### Third-Generation Antipsychotics

Third-generation antipsychotics (TGAs), the newest additions to the antipsychotic class, are characterized by dopamine partial agonism as well as antagonism / weak partial agonism at the 5HT_2A_ receptors (48, 49). Currently there are three approved TGAs, namely aripiprazole, brexpiprazole and cariprazine, often named as the “ABC” drugs (49, 50). Given that these antipsychotics also act on the dopamine D_3_ receptors, which have an important role in cognitive functioning (33), reward and motivation (51), and emotional regulation (52), they are thought to improve not only social dysfunction but negative and affective symptoms of schizophrenia too (53, 54).

Aripiprazole for instance, was found to improve social anxiety in an open-label trial, however the patient number was too small to draw conclusions regarding its efficacy in social dysfunction (55). Regarding brexpiprazole, no study on social dysfunction involving patients with schizophrenia has been conducted, only one animal research was found where dizocilpine-induced social recognition deficits in mice were improved with brexpiprazole treatment (56). As the focus of this perspective is cariprazine, the next section will focus on the available evidence on the role of cariprazine in addressing social dysfunction in schizophrenia.

## Cariprazine, A “Socializing Drug” in the Treatment of Schizophrenia

As mentioned before, cariprazine is a dopamine D_3_/D_2_ and serotonin 5HT_1A_ partial agonist and serotonin 5HT_2A_ antagonist (8). It has a different mechanism of action compared to the other TGAs, as it has the highest affinity for dopamine D_3_ receptors as well as acts at the 5HT_1A_, 5HT_2A_, and α_1B_ receptors too (8, 54). The weakest affinity for the latter is believed to be related to why cariprazine does not induce sedation and hypotension, side effects that commonly bother patients (54). Importantly, the unique D_3_ affinity combined with the action on the different 5HT receptors make cariprazine a potential candidate for addressing those symptoms of schizophrenia that could not be alleviated by previous antipsychotics and therefore were regarded as unmet medical needs.

Cariprazine is currently approved by the Food and Drug Administration (FDA) and European Medicine Agency (EMA) for the treatment of schizophrenia in adults (1.5–6.0 mg/day). In addition, it is approved for the treatment of depressive, acute manic, or mixed episodes associated with bipolar I disorder (3.0–6.0 mg/day) also by the FDA. Furthermore, two Phase III clinical trials found positive results for the adjunctive treatment of major depressive disorder (MDD) with cariprazine^1^. In terms of the schizophrenia indication, the efficacy of cariprazine was demonstrated in three randomized, placebo-controlled Phase II/III clinical trials with patients who had acute exacerbation of schizophrenia (57–59).

### Evidence From Animal and Clinical Trials

In a double-blind, randomized comparative trial cariprazine was found to be superior in treating predominant negative symptoms as measured by the Positive and Negative Syndrome Scale Factor Score for Negative Symptoms (PANSS-FSNS) in patients with schizophrenia compared to an SGA, risperidone (60). Importantly, the results of this trial were repeated in a 16-week, open-label, flexible-dose observational study with 116 schizophrenia patients who also exhibited predominant negative symptoms (61). As there is a strong link between negative symptoms and social dysfunction, these results suggest that cariprazine shows some capacity to sufficiently address social symptoms too.

Indeed, when looking at the Prosocial Functioning Factor that includes the PANSS items N2, N4, N7, P3, P6 and C16 in the above-mentioned trial by Németh and colleagues, change from baseline to week 26 is significantly better with cariprazine than with risperidone (62). Furthermore, a *post-hoc* analysis of one of the Phase III, placebo-controlled studies revealed statistically significant change from baseline to week 6 in the same factor as well (63).

In addition to these results, the Németh study also measured functionality using the Personal and Social Performance (PSP) scale and found that cariprazine significantly improved this aspect from week 10 onwards, again compared to risperidone (60). Importantly, this result was driven by all three relevant subdomains of the PSP scale (60). Finally, in animal research only cariprazine was found to be effective in the social play paradigm when compared to other SGAs (64).

### Evidence From Real-World Experience

Although case reports are not regarded as the highest quality of evidence, they provide personal and specific insight to the effects of a medication (65). In terms of social dysfunction, such descriptions can shed light on the actual impact a drug can induce in real life settings (65). Despite cariprazine is a relatively new antipsychotic on the market, several cases have already been published.

For instance, in a case by Di Sciascio & Palumbo a 22-year-old woman with disorganized schizophrenia was found to improve after switching to cariprazine from olanzapine; she was able to relate to other people again and returned to work (66). Similarly, Halaris & Wuest reported that after a 37-year-old man with a history of chronic psychosis switched to cariprazine he did not only lost a lot of weight spontaneously but also regained his motivation to have a career and live independently. As a result, he started education again and passed the exams successfully (67). Such results were also found in a young female patient suffering from early-onset schizophrenia described by Molnar and colleagues (68). According to their report, the patient was initially socially active but then at the age of 15 she became irritated and physically hostile which ultimately resulted the termination of her studies (68). Soon after cariprazine treatment was initiated, significant improvement in symptoms was observed including starting to participate in the family's daily life (68).

### Safety and Tolerability of Cariprazine

Although the safety and tolerability of an antipsychotic medication is not directly related to how effective it is, they are still important aspects that have indirect impact on the overall outcome. Indeed, as mentioned before, EPS was found to negatively influence social cognition (69). Furthermore, metabolic syndrome, another common side effect of antipsychotic medications, especially SGAs, was found to influence social cognitive performance in patients with schizophrenia (70).

In terms of safety, cariprazine is a safe and generally well tolerated compound (71). The most commonly reported adverse events, according to the pooled analysis of the eight clinical trials with schizophrenia patients, were akathisia, insomnia and headache (71). Importantly, most akathisia was mild or moderate and hence the vast majority of patients remained on treatment (71). In terms of metabolic syndrome, several aspects were measured in the clinical trials including total cholesterol, high-density lipoprotein cholesterol, fasting triglycerides, fasting glucose, weight and body mass index (BMI) (71). Overall, the mean increase from baseline was 1 kg and in general cariprazine was found to be metabolically neutral (71).

## Conclusions

The present perspective aimed at providing an overview of social dysfunction in schizophrenia, its treatment via different generation of antipsychotics and the role of cariprazine in improving such symptoms of the disorder. We argue that based on the reviewed evidence, cariprazine, a potent D_3_ partial agonist can be regarded as a “socializing drug” given its efficacy in treating negative, cognitive, and affective symptoms that has been proven in animal research, clinical trials, an observational study, as well as in individual cases. We understand that the reviewed evidence is limited in a sense that no study has been conducted to specifically measure the efficacy of cariprazine in improving social dysfunction and hence encourage further research to investigate this aspect in a meaningful design.

## Data Availability Statement

The original contributions presented in the study are included in the article/supplementary material, further inquiries can be directed to the corresponding author.

## Author Contributions

PM, RB, and YB conceptualized the perspective. PM prepared the first draft which was reviewed by RB and YB. All authors contributed to the article and approved the submitted version.

## Funding

The manuscript has been written independently. Recordati S.p.A agreed to provide the open access fee for this manuscript.

## Conflict of Interest

The authors declare that the research was conducted in the absence of any commercial or financial relationships that could be construed as a potential conflict of interest.

## Publisher's Note

All claims expressed in this article are solely those of the authors and do not necessarily represent those of their affiliated organizations, or those of the publisher, the editors and the reviewers. Any product that may be evaluated in this article, or claim that may be made by its manufacturer, is not guaranteed or endorsed by the publisher.

## Footnotes

^1^ https://www.gedeonrichter.com/en/news/211029

## References

[1] WHO. WHO | Schizophrenia. Schizophrenia. 2018.

[2] Bleuler E. Dementia Praecox or the Group of Schizophrenias. Translated by Joseph Zinkin. Int Univ Press (1950)

[3] Galderisi S Mucci A Buchanan RW Arango C. Negative symptoms of schizophrenia: new developments and unanswered research questions. Lancet Psychiatry. (2018) 5:664–77. 10.1016/S2215-0366(18)30050-6 29602739

[4] Bowie CR Harvey PD. Cognitive deficits and functional outcome in schizophrenia. Neuropsychiatr Dis Treat. (2006) 2:531–6. 10.2147/nedt.2006.2.4.531 19412501 PMC2671937

[5] Harvey PD Strassnig M. Predicting the severity of everyday functional disability in people with schizophrenia: cognitive deficits, functional capacity, symptoms, and health status. World Psychiatry. (2012) 11:73–9. 10.1016/j.wpsyc.2012.05.004 22654932 PMC3363376

[6] Charlson FJ Ferrari AJ Santomauro DF Diminic S Stockings E Scott JG. Global epidemiology and burden of schizophrenia: Findings from the global burden of disease study 2016. Schizophr Bull. (2018) 44:1195–203. 10.1093/schbul/sby058 29762765 PMC6192504

[7] Torres-González F Ibanez-Casas I Saldivia S Ballester D Grandón P Moreno-Küstner B. Unmet needs in the management of schizophrenia. Neuropsychiatr Dis Treat. (2014) 10:97–110. 10.2147/NDT.S41063 24476630 PMC3897352

[8] Stahl SM. Mechanism of action of cariprazine. CNS Spectr. (2016) 21:123–7. 10.1017/S1092852916000043 26956157

[9] Bon J Repovš G Pileckyte I Škodlar B. Variable causes of social dysfunction in schizophrenia: the interplay of neurocognitive, personal, and intersubjective factors. Anthropol Notebooks. (2016) 22:5–30.

[10] Kendler KS. Kraepelin's final views on dementia praecox. Schizophr Bull. (2021) 47:635–43. 10.1093/schbul/sbaa177 33320201 PMC8673439

[11] Kraepelin E. Einführung in die Psychiatrische Klinik. J Nerv Ment Dis. (1922) 55:74. 10.1097/00005053-192201000-00041

[12] American Psychiatric Association. American Psychiatric Association: Diagnostic and Statistical Manual of Mental Disorders. Fifth Edition. Arlington (2013).

[13] Green MF Kern RS Heaton RK. Longitudinal studies of cognition and functional outcome in schizophrenia: implications for MATRICS. Schizophr Res. (2004) 72:41–51. 10.1016/j.schres.2004.09.009 15531406

[14] Sergi MJ Rassovsky Y Nuechterlein KH Green MF. Social perception as a mediator of the influence of early visual processing on functional status in schizophrenia. Am J Psychiatry. (2006) 163:448–54. 10.1176/appi.ajp.163.3.448 16513866

[15] Fett AKJ Viechtbauer W Dominguez M de G Penn DL van Os J Krabbendam L. The relationship between neurocognition and social cognition with functional outcomes in schizophrenia: a meta-analysis. Neurosci Biobehav Rev. (2011) 35:573–88. 10.1016/j.neubiorev.2010.07.001 20620163

[16] Robertson BR Prestia D Twamley EW Patterson TL Bowie CR Harvey PD. Social competence versus negative symptoms as predictors of real world social functioning in schizophrenia. Schizophr Res. (2014) 160:136–41. 10.1016/j.schres.2014.10.037 25468184 PMC4258126

[17] Marder SR Galderisi S. The current conceptualization of negative symptoms in schizophrenia. World Psychiatry. (2017) 16:14–24. 10.1002/wps.20385 28127915 PMC5269507

[18] Kasanova Z Oorschot M Myin-Germeys I. Social anhedonia and asociality in psychosis revisited. An experience sampling study. Psychiatry Res. (2018) 270:375–81. 10.1016/j.psychres.2018.09.057 30300867

[19] Desalegn D Girma S Abdeta T. Quality of life and its association with psychiatric symptoms and socio-demographic characteristics among people with schizophrenia: a hospital-based cross-sectional study. PLoS One. (2020) 15:e0229514. 10.1371/journal.pone.0229514 32092123 PMC7039432

[20] Tsai J Stroup TS Rosenheck RA. Housing arrangements among a national sample of adults with chronic schizophrenia living in the United States: a descriptive study. J Community Psychol. (2011) 39:76–88. 10.1002/jcop.20418

[21] Ang MS Rekhi G Lee J. Associations of living arrangements with symptoms and functioning in schizophrenia. BMC Psychiatry. (2021) 21:497. 10.1186/s12888-021-03488-5 34635064 PMC8507381

[22] Evensen S Wisløff T Lystad JU Bull H Ueland T Falkum E. Prevalence, employment rate, and cost of schizophrenia in a high-income welfare society: a population-based study using comprehensive health and welfare registers. Schizophr Bull. (2016) 42:476−83. 10.1093/schbul/sbv141 26433216 PMC4753607

[23] Holm M Taipale H Tanskanen A Tiihonen J Mitterdorfer-Rutz E. Employment among people with schizophrenia or bipolar disorder: a population-based study using nationwide registers. Acta Psychiatr Scand. (2021) 143:61–71. 10.1111/acps.13254 33155273 PMC7839734

[24] Hakulinen C McGrath JJ Timmerman A Skipper N Mortensen PB Pedersen CB. The association between early-onset schizophrenia with employment, income, education, and cohabitation status: nationwide study with 35 years of follow-up. Soc Psychiatry Psychiatr Epidemiol. (2019) 54:1343–51. 10.1007/s00127-019-01756-0 31456027

[25] Ranning A Laursen T Agerbo E Thorup A Hjorthoj C Jepsen JRM. School performance from primary education in the adolescent offspring of parents with schizophrenia and bipolar disorder-a national, register-based study. Psychol Med. (2018) 48:1993–2000. 10.1017/S0033291717003518 29239287

[26] Samuel R Thomas E Jacob KS. Instrumental activities of daily living dysfunction among people with schizophrenia. Indian J Psychol Med. (2018) 16:29–35. 10.4103/IJPSYM.IJPSYM_308_17 29962569 PMC6008996

[27] Stain HJ Galletly CA Clark S Wilson J Killen EA Anthes L. Understanding the social costs of psychosis: the experience of adults affected by psychosis identified within the second Australian national survey of psychosis. Aust N Z J Psychiatry. (2012) 46:879–89. 10.1177/0004867412449060 22645395

[28] Morgan VA Waterreus A Jablensky A MacKinnon A McGrath JJ Carr V. People living with psychotic illness in 2010: the second Australian national survey of psychosis. Aust N Z J Psychiatry. (2012) 10.1037/e505512012-001 22696547

[29] Cacioppo JT Hawkley LC. Perceived social isolation and cognition. Trends Cogn Sci. (2009) 13:447–54. 10.1016/j.tics.2009.06.005 19726219 PMC2752489

[30] Brunet-Gouet E Decety J. Social brain dysfunctions in schizophrenia: a review of neuroimaging studies. Psychiatry Res - Neuroimaging. (2006) 148:75–92. 10.1016/j.pscychresns.2006.05.001 17088049

[31] Thakkar KN Peterman JS Park S. Altered brain activation during action imitation and observation in schizophrenia: a translational approach to investigating social dysfunction in schizophrenia. Am J Psychiatry. (2014) 171:539–48. 10.1176/appi.ajp.2013.13040498 24626638 PMC4578626

[32] Bäckman L Nyberg L Lindenberger U Li SC Farde L. The correlative triad among aging, dopamine, and cognition: current status and future prospects. Neurosci Biobehav Rev. (2006) 30:791–807. 10.1016/j.neubiorev.2006.06.005 16901542

[33] Nakajima S Gerretsen P Takeuchi H Caravaggio F Chow T Le Foll B. The potential role of dopamine D3 receptor neurotransmission in cognition. Eur Neuropsychopharmacol. (2013) 23:799–813. 10.1016/j.euroneuro.2013.05.006 23791072 PMC3748034

[34] Krakowski M. Violence and serotonin: Influence of impulse control, affect regulation, and social functioning. J Neuropsychiatry Clin Neurosci. (2003) 15:294–305. 10.1176/jnp.15.3.294 12928505

[35] Vartiainen H Tiihonen J Putkonen A Koponen H Virkkunen M Hakola P. Citalopram, a selective serotonin reuptake inhibitor, in the treatment of aggression in schizophrenia. Acta Psychiatr Scand. (1995) 91:348–51. 10.1111/j.1600-0447.1995.tb09793.x 7639092

[36] Kucharska-Pietura K Mortimer A. Can antipsychotics improve social cognition in patients with schizophrenia? CNS Drugs. (2013) 27:335–43. 10.1007/s40263-013-0047-0 23533009 PMC3657085

[37] Stahl SM. Stahl's essential psychopharmacology: neuroscientific basis and practical applications. 4th Edition. Cambridge University Press (2013).

[38] Kapur S Remington G. Atypical antipsychotics: new directions and new challenges in the treatment of schizophrenia. Annu Rev Med. (2001) 52:503–17. 10.1146/annurev.med.52.1.503 11160792

[39] Hill SK Bishop JR Palumbo D Sweeney JA. Effect of second-generation antipsychotics on cognition: current issues and future challenges. Expert Rev Neurother. (2010) 10:43–57. 10.1586/ern.09.143 20021320 PMC2879261

[40] Newcomer JW. Second-generation (atypical) antipsychotics and metabolic effects: a comprehensive literature review. CNS Drugs. (2005) 19(Suppl. 1):1–93. 10.2165/00023210-200519001-00001 15998156

[41] Rummel-Kluge C Komossa K Schwarz S Hunger H Schmid F Kissling W. Second-generation antipsychotic drugs and extrapyramidal side effects: a systematic review and meta-analysis of head-to-head comparisons. Schizophr Bull. (2012) 38:167–77. 10.1093/schbul/sbq042 20513652 PMC3245581

[42] Bellack AS Schooler NR Marder SR Kane JM Brown CH Yang Y. Do clozapine and risperidone affect social competence and problem solving. Am J Psychiatry. (2004) 161:364–7. 10.1176/appi.ajp.161.2.364 14754789

[43] Sergi MJ Green MF Widmark C Reist C Erhart S Braff DL. Cognition and neurocognition: effects of risperidone, olanzapine, and haloperidol. Am J Psychiatry. (2007) 164:1585–92. 10.1176/appi.ajp.2007.06091515 22688151

[44] Harvey PD Patterson TL Potter LS Zhong K Brecher M. Improvement in social competence with short-term atypical antipsychotic treatment: a randomized, double-blind comparison of quetiapine versus risperidone for social competence, social cognition, and neuropsychological functioning. Am J Psychiatry. (2006) 163:1918–25. 10.1176/ajp.2006.163.11.1918 17074943

[45] Penn DL Keefe RSE Davis SM Meyer PS Perkins DO Losardo D. The effects of antipsychotic medications on emotion perception in patients with chronic schizophrenia in the CATIE trial. Schizophr Res. (2009) 115:17–23. 10.1016/j.schres.2009.08.016 19766459 PMC2765056

[46] Fakra E Salgado-Pineda P Besnier N Azorin JM Blin O. Risperidone vs. haloperidol for facial affect recognition in schizophrenia: Findings from a randomised study. World J Biol Psychiatry. (2009) 10:719–28. 10.1080/15622970701432536 17853271

[47] Qiao H Noda Y Kamei H Nagai T Furukawa H Miura H. Clozapine, but not haloperidol, reverses social behavior deficit in mice during withdrawal from chronic phencyclidine treatment. Neuroreport. (2001) 12:11–5. 10.1097/00001756-200101220-00010 11201068

[48] Mailman R Murthy V. Third generation antipsychotic drugs: partial agonism or receptor functional selectivity? Curr Pharm Des. (2010) 16:488–501. 10.2174/138161210790361461 19909227 PMC2958217

[49] Cheng J Wang S. Structure-based design of a novel third-generation antipsychotic drug lead with potential antidepressant properties. Nat Neurosci. (2022) 25:39–49. 10.1038/s41593-021-00971-w 34887590

[50] Citrome L. The ABC's of dopamine receptor partial agonists - aripiprazole, brexpiprazole and cariprazine: the 15-min challenge to sort these agents out. Int J Clin Pract. (2015) 69:1211–20. 10.1111/ijcp.12752 26477545

[51] Wise RA. Brain reward circuitry: Insights from unsensed incentives. Neuron. (2002) 36:229–40. 10.1016/S0896-6273(02)00965-0 12383779

[52] Cortés A Moreno E Rodríguez-Ruiz M Canela EI Casadó V. Targeting the dopamine D3 receptor: an overview of drug design strategies. Expert Opin Drug Discov. (2016) 11:641–64. 10.1080/17460441.2016.1185413 27135354

[53] Gross G Drescher K. The role of dopamine D 3 receptors in antipsychotic activity and cognitive functions. Handb Exp Pharmacol. (2012) 213:167–210. 10.1007/978-3-642-25758-2_7 23027416

[54] Frankel JS Schwartz TL. Brexpiprazole and cariprazine: distinguishing two new atypical antipsychotics from the original dopamine stabilizer aripiprazole. Ther Adv Psychopharmacol. (2017) 7:29–41. 10.1177/2045125316672136 28101322 PMC5228714

[55] Stern RG Petti TA Bopp K Tobia A. Aripiprazole for the treatment of schizophrenia with co-occurring social anxiety: an open-label cross-taper study. J Clin Psychopharmacol. (2009) 29:206–9. 10.1097/JCP.0b013e3181a48e12 19440071

[56] Yoshimi N Futamura T Hashimoto K. Improvement of dizocilpine-induced social recognition deficits in mice by brexpiprazole, a novel serotonin-dopamine activity modulator. Eur Neuropsychopharmacol. (2015) 25:356–64. 10.1016/j.euroneuro.2014.12.014 25600995

[57] Durgam S Starace A Li D Migliore R Ruth A Németh G. An evaluation of the safety and efficacy of cariprazine in patients with acute exacerbation of schizophrenia: a phase II, randomized clinical trial. Schizophr Res. (2014) 152:450–7. 10.1016/j.schres.2013.11.041 24412468

[58] Durgam S Cutler AJ Lu K Migliore R Ruth A Laszlovszky I. Cariprazine in acute exacerbation of schizophrenia: a fixed-dose, phase 3, randomized, double-blind, placebo- and active-controlled trial. J Clin Psychiatry. (2015) 76:e1574–82. 10.4088/JCP.15m09997 26717533

[59] Kane JM Zukin S Wang Y Lu K Ruth A Nagy K. Efficacy and safety of cariprazine in acute exacerbation of schizophrenia: results from an international, phase III clinical trial. J Clin Psychopharmacol. (2015) 35:367–73. 10.1097/JCP.0000000000000346 26075487

[60] Németh G Laszlovszky I Czobor P Szalai E Szatmári B Harsányi J. Cariprazine vs. risperidone monotherapy for treatment of predominant negative symptoms in patients with schizophrenia: a randomised, double-blind, controlled trial. Lancet. (2017) 389:1103–13. 10.1016/S0140-6736(17)30060-0 28185672

[61] Rancans E Dombi ZB Mátrai P Barabaissy A Sebe B Skrivele I. The effectiveness and safety of cariprazine in schizophrenia patients with negative symptoms and insufficient effectiveness of previous antipsychotic therapy: an observational study. Int Clin Psychopharmacol. (2021) 36:154–61 10.1097/YIC.0000000000000351 33560040 PMC8011502

[62] Fleischhacker W Galderisi S Laszlovszky I Szatmári B Barabássy Á Acsai K. The efficacy of cariprazine in negative symptoms of schizophrenia: *Post hoc* analyses of PANSS individual items and PANSS-derived factors. Eur Psychiatry. (2019) 58:1–9. 10.1016/j.eurpsy.2019.01.015 30738380

[63] Daniel D Nasrallah H Earley W Durgam S Lu K Szatmári B. 17. Effects of cariprazine on negative symptoms, cognitive impairment, and prosocial functioning in patients with predominant negative symptoms: *post hoc* analysis of a phase III, placebo-, and active-controlled study. Schizophr Bull. (2017) 43(Issue suppl_1):S13. 10.1093/schbul/sbx021.036

[64] Román V Adham N Foley AG Hanratty L Farkas B Lendvai B. Cariprazine alleviates core behavioral deficits in the prenatal valproic acid exposure model of autism spectrum disorder. Psychopharmacology. (2021) 238:2381–f2393. 10.1007/s00213-021-05851-6 34264367 PMC8373751

[65] Radawski CA Hammad TA Colilla S Coplan P Hornbuckle K Freeman E. The utility of real-world evidence for benefit-risk assessment, communication, and evaluation of pharmaceuticals: case studies. Pharmacoepidemiol Drug Saf . (2020) 29:1532–9. 10.1002/pds.5167 33146901

[66] di Sciascio G Palumbo C. Experiences of switching to cariprazine. Evid based Psyxhiatric Care. (2019) 5(Supplemento 03):8–10.

[67] Halaris A Wuest J. Metabolic syndrome reversal with cariprazine. J Clin Psychopharmacol. (2019) 39:413–6. 10.1097/JCP.0000000000001074 31188240

[68] Molnar MJ Jimoh IJ Zeke H Palásti Á Fedor M. Early-onset schizophrenia with predominantly negative symptoms: a case study of a drug-naive female patient treated with cariprazine. Front Pharmacol. (2020) 11:477. 10.3389/fphar.2020.00477 32390838 PMC7191004

[69] Monteleone P Cascino G Monteleone AM Rocca P Rossi A Bertolino A. Prevalence of antipsychotic-induced extrapyramidal symptoms and their association with neurocognition and social cognition in outpatients with schizophrenia in the “real-life.” Prog Neuro-Psychopharmacology Biol Psychiatry. (2021) 109:110250. 10.1016/j.pnpbp.2021.110250 33484755

[70] Chen S Xia X Deng C Wu X Han Z Tao J. The correlation between metabolic syndrome and neurocognitive and social cognitive performance of patients with schizophrenia. Psychiatry Res. (2020) 288:112941. 10.1016/j.psychres.2020.112941 32334274

[71] Barabássy Á Sebe B Acsai K Laszlovszky I Szatmári B Earley WR. Safety and tolerability of cariprazine in patients with schizophrenia: a pooled analysis of eight phase ii/iii studies. Neuropsychiatr Dis Treat. (2021) 17:957–70. 10.2147/NDT.S301225 33854317 PMC8040316

